# Hepatitis C virus screening in community pharmacies: results on feasibility from a Swiss pilot

**DOI:** 10.1186/s12879-023-08362-1

**Published:** 2023-06-07

**Authors:** Dominik Stämpfli, Tamara Leila Imfeld-Isenegger, Kurt E. Hersberger, Markus Messerli

**Affiliations:** 1 Institute of Pharmaceutical Sciences, Department of Chemistry and Applied Biosciences, Swiss Federal Institute of Technology, ETH Zurich, Vladimir-Prelog-Weg 4, CH-8093 Zurich, Switzerland; 2 Hospital Pharmacy, Kantonsspital Baden, Switzerland; 3 Pharmaceutical Care Network Switzerland, Basel, Switzerland; 4 Hospital Pharmacy, Luzerner Kantonsspital, Luzern, Switzerland; 5 Pharmaceutical Care Research Group, Department of Pharmaceutical Sciences, University of Basel, Basel, Switzerland

**Keywords:** Hepatitis C, HCV, Community Pharmacy Services, Saliva, Vulnerable

## Abstract

**Background:**

Hepatitis C virus (HCV) infections are a public health burden worldwide and often go undetected until sequelae develop. Offering HCV screening for the different vulnerable populations in community pharmacies could help prevent further undetected HCV infections. This pilot aimed to assess the feasibility and pharmacist acceptance of HCV rapid antibody saliva testing in community pharmacies.

**Methods:**

A structured pharmaceutical care intervention was developed that included addressing, informing, and screening clients, as well as referral and reporting to subsequent health care providers. Participating pharmacies from French-, German- and Italian-speaking parts of Switzerland were trained to provide this service to local vulnerable populations. Information on client recruitment, feasibility, and acceptability of HCV screening was collected.

**Results:**

Of 36 pharmacies initially recruited, 25 started the pilot and approached 435 clients, 145 of whom (33%) were interested in screening. Eight of these rapid antibody tests returned positive (prevalence rate: 5.5%). Facilitators were being able to offer a free rapid test (73%), followed by having training prior to the project (67%) and having a new service to offer (67%). The possibility of clients reacting dismissively (53%) and of unsettling clients (47%) were reported to be the main barriers.

**Conclusions:**

This pilot demonstrated the general feasibility of an HCV screening service with rapid antibody saliva testing in Swiss community pharmacies, which achieved a higher prevalence rate than national estimates. With appropriate communication training and remuneration, Swiss community pharmacies could be an important partner in implementing HCV elimination strategies.

## Introduction

Hepatitis-C-virus (HCV) infections are a worldwide public health burden owing to their morbidity and mortality [1]. The World Health Organization (WHO) estimates that in 2019, 58 million people were living with an HCV infection, causing 290 000 deaths [2]. Acute infections can be accompanied by rather unspecific symptoms such as fatigue, difficulty concentrating, nausea, abdominal pain and arthralgia, but can also remain asymptomatic until the onset of sequelae [3, 4]. After six months, 50–85% of patients do not clear the virus and the infections evolve into a chronic HCV infection [5]. Subsequently, up to 50% of chronic HCV patients develop liver cirrhosis over the course of 10 to 20 years [6–8], and approximately 25% of hepatocellular carcinoma cases are attributable to HCV [9]. Fortunately, direct acting antiviral drugs are now available that can lead to complete viral elimination with sustained virological response. Therefore, global control or even elimination of chronic HCV seems in reach [10].

Estimations for Switzerland indicated in 2016 that around 0.5% (40 000 Swiss citizens) are HCV carriers [11], of which approximately one third are undetected [12]. Every year, around 200 people die in Switzerland as a result of an HCV infection [13]. Due to the low prevalence of HCV in the general Swiss population, the Swiss Federal Office of Public Health has decided against a national population screening to identify unnoticed HCV infections. Rather, specific vulnerable populations should be targeted for testing. Various studies specified groups with an increased risk for the occurrence of HCV infections in Switzerland, including intravenous drug users, birth cohorts 1955–1974, recipients of blood, blood products or organs before 1992, persons immigrated from HCV endemic regions, and men having sex with men [11, 14–17]. In a recent publication, Bihl and colleagues discussed the current HCV disease burden in Switzerland and concluded that additional efforts are needed for diagnosis and care uptake, and that further interventions, including increased provider coverage and more intensive screening, may identify patients who otherwise fall through gaps in care or avoid care due to stigma. Their findings suggest that the annual number of newly diagnosed and treated patients is declining, threatening Switzerland's progress towards HCV elimination by 2030 [18].

In its guidance on Human Immunodeficiency Virus, hepatitis B and C testing, the European Centre for Disease Prevention and Control stated that testing needs to be provided through community pharmacies as well [19]. The opportunities from HCV testing and treatments in community pharmacies have been described in studies from the USA, New Zealand, and the UK, with the National Health Service England now commissioning a national community pharmacy-based testing programme, and a consensus statement on the implementation of testing services being in development [20]. Examples of studies include the dry blood spot testing service in community pharmacies on the Isle of Wight, in which 20 pharmacies performed around 200 tests over a two-year period, which identified 13 new cases of HCV [21]. In 2020, Radley and colleagues published their results on a Scottish cluster-randomised trial, which allocated pharmacies to either offer pharmacist-led care to opioid substitution users, including HCV treatment initiation and continuation, or conventional care with a nurse prescriber in a treatment centre [22]. With 98 of 1365 patients (7%) in the pharmacist-led care group compared to 43 of 1353 patients (3%) in the conventional care group, significantly more patients had a sustained virological response after 12 weeks. This study showcased the benefit of having pharmacists, who are in contact with opioid substitution users anyways (e.g., supply of the daily methadone dose), involved in the entire process of diagnosis and treatment of HCV. Likewise, Hunt and colleagues described a pharmacist-led HCV programme in Chicago, which involves (clinical) pharmacists reviewing patient records, selecting treatment regimens, acquiring insurance approval, educating patients on adherence, and monitoring treatment effectiveness within a collaborative team at an infectious disease clinic. Here, pharmacists effectively led patients through the treatment cascade after a universal screening programme [23]. Similarly, Downes and colleagues described the effectiveness of clinical pharmacist-led HCV treatment management at a health centre in Omaha. Whilst 9.9% of patients with a positive HCV viral load initiated treatment in the usual care group, 57.1% were referred to treatment though the pharmacist-led management [24]. These examples display an opportunity for pharmacists to practice pharmaceutical care for patients with a positive HCV viral load, a "contribution to the care of individuals in order to optimise medicines use and improve health outcomes" [25].

The independent association Pharmaceutical Care Network Switzerland (PCN-S, www.pcn-s.ch), which aims to promote pharmaceutical care in Switzerland, identified Radley and colleagues' work on HCV detection as a potential service for Swiss pharmacies and vulnerable populations. Out of members of PCN-S, a study group comprising the four authors initiated the pilot project "Hepatitis C Screening in Swiss Community Pharmacies", offering low-threshold saliva testing at no cost for vulnerable populations for HCV as specified by the Swiss Federal Office of Public Health [13, 17]. The project was developed in collaboration with the Swiss Hepatitis Association (https://en.hepatitis-schweiz.ch), a private multi-stakeholder network.

### Aims

Based on the Medical Research Council framework for developing and evaluating complex interventions [26], our aim was to assess the feasibility and pharmacist acceptance of this pilot HCV screening service with rapid antibody saliva testing for vulnerable populations in community pharmacies.

## Methods

### Intervention

**Fig. 1:**
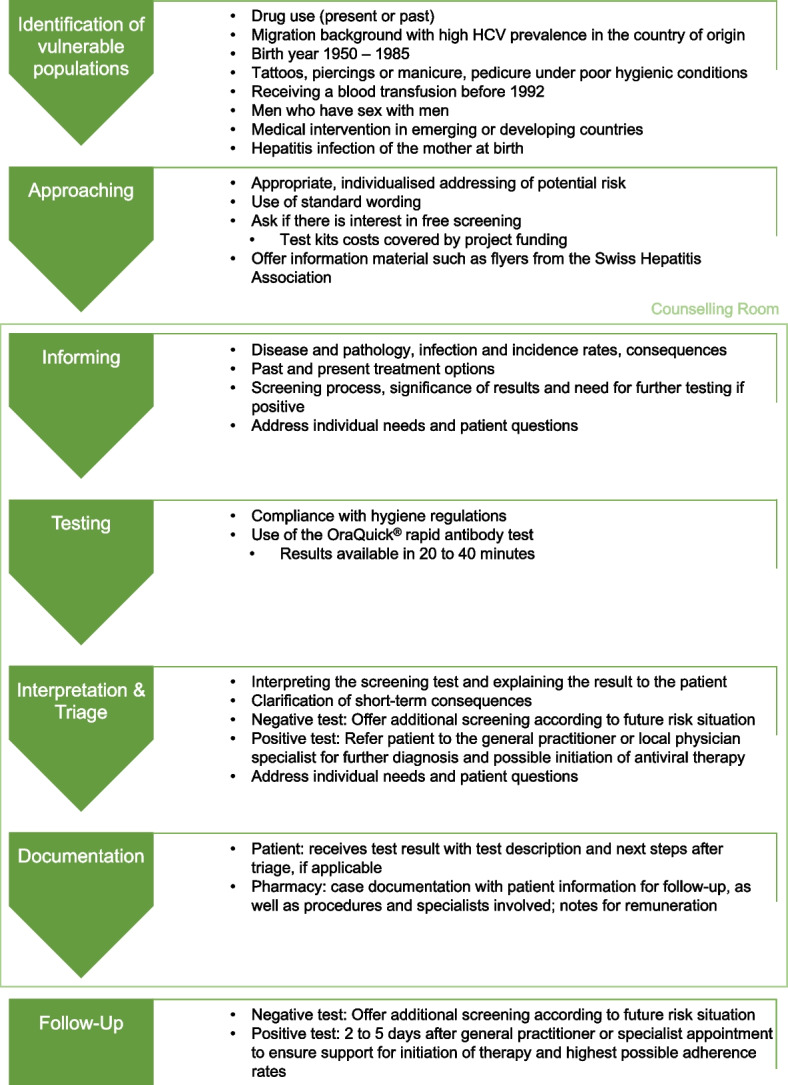
Screening procedure for hepatitis C infections in Swiss community pharmacies

The independent association Pharmaceutical Care Network Switzerland (PCN-S) developed a structured process to ensure a safe and effective screening procedure (Fig. 1).

Pharmacists were first trained through i) an online lecture on the pathology, incidence rates, and vulnerable populations, ii) a webinar on the developed screening intervention, including the use of the saliva test, and iii) an on-site training in the pharmacy focusing on the individual strategy and communication for recruiting clients from two vulnerable populations, as defined by the Swiss Federal Office of Public Health. Each element of the process (Fig. 1) was explored in the webinar and on-site training, and its relevance to targeting and screening was highlighted. Each pharmacy individually decided which vulnerable populations to address, considering the different population groups and clients’ profile based on their local circumstances. The trained pharmacy staff agreed on an individual approach strategy with a tailored introduction and explanation of the opportunity for screening. Their specific approach was then practiced in role play with feedback discussions.

Clients who consented to the screening were informed about the exact procedure in a separate room. A saliva sample was collected for the OraQuick^®^ rapid antibody test device [27, 28]. Test results were available within 20 to 40 min. In case of a positive test, clients were informed that a more invasive diagnostic procedure was needed to determine the exact infection status and to initiate drug therapy. The pharmacy then contacted the client's primary care physicians and informed them about the screening intervention performed. A referral letter was written with information on how to proceed with the diagnosis and contact information on local physician specialists to ensure interprofessional and interdisciplinary cooperation. Pharmacies were asked to make a follow-up call within one week of screening to ensure completion of the diagnostic process and support successful initiation of drug therapy.

Pharmacists were recruited in French- (F-CH), German- (D-CH) and Italian-speaking (I-CH) parts of Switzerland. In D-CH, PCN-S conducted the recruitment and used its network to motivate independent and chain-pharmacies to participate in the pilot study. In F-CH and I-CH, recruitment was organised by the customer care department of Abbvie Lt Switzerland. Costs for the test kits were covered by the project funding (approx. 22 US-Dollars per test). Additionally, a reimbursement to the pharmacies was paid for each customer recruited and screened (approx. 70 US-Dollars). The pharmacies reported the number of screenings back to the study group on their own responsibility, which was used to calculate the pay-outs.

### Feasibility outcomes

Inspired by the implementation outcomes by Proctor and colleagues [29], we assessed *acceptability* (perception that the service is agreeable, palatable, or satisfactory), *adoption* (intention or action to try or employ the service), *appropriateness* (perceived fit of the service for the provider), and *feasibility* (extent to which the service can be successfully carried out within the given setting) of performing the HCV screening in community pharmacies.

Adoption was assessed by asking community pharmacies to report their aggregated counts of reasons for addressing (i.e., approaching) and recruiting clients (i.e., conducting rapid test) according to the clients’ vulnerability [13, 17] with a questionnaire once per pharmacy. If the clients were part of two or more vulnerable populations, we asked the pharmacies to only count the primary reason for addressing the client. We allowed for submission of additional, free text reasons. To protect the individual vulnerable populations, only the total number of positive rapid tests were reported to the study group as part of the pay-outs, without specifying the reason for initially addressing the clients and, hence, exposing the vulnerable population.

Acceptability, appropriateness, and feasibility were measured by inviting each pharmacist, who actively participated in the pilot, to rate the eight items “I think hepatitis C screening is a useful service for our customers” (acceptability), “Would you continue to offer the service?” (acceptability), “Would you participate in such a pilot project again?” (acceptability), “I think that hepatitis C screening fits into the services offered by our pharmacy” (appropriateness), “Our customers want to use hepatitis C screening when I approach them about the offer” (appropriateness), “Hepatitis C screening was easy to integrate into my workflow” (feasibility), “I found the OraQuick tests easy to use” (feasibility), and “You quickly learn to work with the OraQuick test” (feasibility) on a 6-point Likert scale from “does not apply at all” to “does fully apply.” We also asked about barriers and facilitators: “What were the challenges for you in this project?” and “What were favourable factors for you?”. These were multi-selectable yes/no-items that were pre-defined by the authors, as well as individual free-text responses. Respondents were also asked to report on highlights and weaknesses of the project.

The questionnaires, available in German and French, were generated with SelectSurvey.NET version 5 (ClassApps, Kansas City, MO 64108 USA) with servers located within Switzerland at the Swiss Federal Institute of Technology, ETH Zurich.

### Statistical analysis

We performed descriptive analyses on both questionnaires, reporting on frequencies. We used RStudio, version 1.3.1093, running R, version 4.0.2 [30], for our analyses. Additional packages included tidyverse, dplyr, data.table, and ggplot2.

## Results

### Setting

The structured process was developed in February 2021, pharmacy recruitment and staff training took place in March 2021, and implementation of the screening intervention in daily practice was conducted from April to September 2021. Of 36 participating pharmacies, 25 actively implemented the HCV screening (dropout rate: 31%). These 25 pharmacies reported having approached 435 customers, of whom 145 agreed to be screened (33%). In F-CH, 5 pharmacies approached 170 customers and conducted 45 screenings (number needed to approach for screening, NNA: 3.8). In D-CH, 7 pharmacies approached 127 customers and carried out 26 screenings (NNA: 4.9). And in I-CH, 13 pharmacies approached 138 customers and carried out 74 screenings (NNA: 1.9). By reporting back to the study group for pay-outs, pharmacies stated on having screened 145 clients, of which eight (5.5%) generated a positive test.

### Adoption

By questionnaire, 15 community pharmacies (60%) reported on having addressed a total of 257 clients, of which 79 (30.7%) were recruited for conducting the rapid test. Table 1 shows the distribution and recruitment rates across the different HCV vulnerable populations. The three most addressed populations were history of or active drug use (75), history of migration (60), and birth year between 1950 and 1985 (51). Additional, free text reasons for addressing and recruiting clients were: General symptoms (e.g., fatigue), infection with the human immunodeficiency virus (HIV), needle accidents, sexual orientation, and dialysis. The highest rate of recruitment had tattoos and piercing, with 14 of 25 addressed clients (56.0%) conducting a rapid test.

**Table 1:** Distribution and recruitment rates for hepatitis C screening in community pharmacies among different vulnerable populations

Vulnerability^a^	Addressed	Recruited for rapid test (% of addressed)
Drug use (injecting or sniffing drugs)	75	26 (34.7)
Migration background	60	5 (8.3)
Birth year 1950—85	51	23 (45.1)
Tattoos, piercings or manicure, pedicure under poor hygienic conditions	25	14 (56.0)
Receiving blood transfusion before 1992	12	1 (8.3)
Medical procedure in emerging or developing countries	9	0 (0)
Hepatitis infection of the mother at birth	3	0 (0)
Others	22	10 (45.5)
Total	257	79 (30.7)

^a^adapted from [13, 17]

### Acceptability, appropriateness, and feasibility

**Fig. 2:**
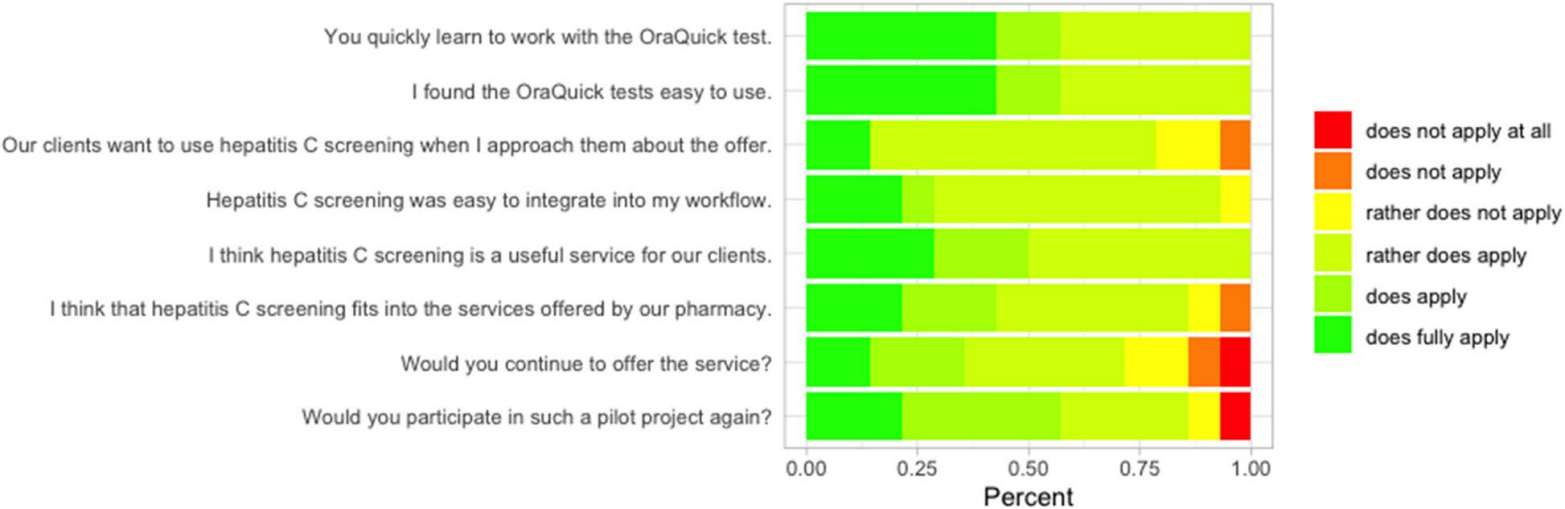
Ratings of 15 participating pharmacists on the acceptability, appropriateness, and feasibility of hepatitis C screenings in community pharmacies. Six-point Likert scale

Our questionnaire focusing on acceptability, appropriateness, and feasibility was answered by a total of 15 community pharmacists (10 through PCN-S, five individual), with six from the D-CH part, eight from the I-CH, and one from the F-CH part of Switzerland. Figure 2 visualises their ratings on the 6-point Likert scale. The respondents’ age averaged at 39 years (standard deviation [SD] =  ± 12 years), and their work experience at 13 years (SD = 12 years). Concerning acceptability, 71.4% (*n* = 10) pharmacists wanted to continue the service, whilst 28.5% [4] would not continue. Concerning appropriateness, 78.6% [11] of the responding pharmacists stated that their clients wanted to use the screening when approached (64.3% rather does apply, 14.3% does fully apply). Asked on feasibility, only 7.1% [1] stated that the screening was not easy to integrate into the workflow.

Frequencies of barriers that were queried as pre-defined yes/no-items are shown in Table 2. With 53.3%, most pharmacists stated that clients, that were approached, could react dismissively. This barrier was followed by the possibility of unsettling the client when approaching about the service (46.7%) and the general workload (26.7%). Additional individual, free text barriers that were mentioned concerned HCV as a topic (being a taboo, clients being overwhelmed), personnel resources being bound for around 30 min, and clients not following up on an appointment.

**Table 2:** Frequency of hepatitis C screening barriers in community pharmacies surveyed as pre-defined yes/no-items. *N* = 15

Barriers	N (%)
Clients approached react dismissively	8 (53.3)
Unsettling the client when approaching about the service	7 (46.7)
General workload	4 (26.7)
Insecurities with the topic	2 (13.3)
Insecurities with handling the rapid test	1 (6.7)
Insecurities with handling potentially infectious material	0 (0)
Too little support from the pharmacy	0 (0)
Complicated process	0 (0)
Problems with the process	0 (0)
Service requires too much effort	0 (0)
Too little preparation time	0 (0)

Frequencies of facilitators that were queried as pre-defined yes/no-items are shown in Table 3. With 73.3%, most pharmacists stated that being able to offer a free rapid test was the primary facilitator in offering an HCV screening. This was followed by having an extensive training prior to the project (66.7%) and having a new service to offer (66.7%). Additional individual, free text facilitators included the opportunity of offering a service free-of-charge and the project being used as thesis for a further education course.

**Table 3:** Frequency of hepatitis C screening facilitators in community pharmacies surveyed as pre-defined yes/no-items. *N* = 15

Facilitator	N (%)
Free rapid tests	11 (73.3)
Training	10 (66.7)
New service	10 (66.7)
Available documentation	6 (40.0)
Remuneration	6 (40.0)
Coaching	4 (26.7)
Flyer	4 (26.7)
Client retention	4 (26.7)
Helpline	2 (13.3)
Online information repository	0 (0)

Community pharmacists stated additional topics within the free text highlights and weaknesses concerning the pilot in general, shown in Table 4. Highlights mainly included positive interactions with their clients and being able to offer free-of-charge rapid antibody saliva tests. Weaknesses majorly included the time investment and issues surrounding the sensitive topic.

**Table 4:** Highlights and weaknesses concerning the project as stated by the responding pharmacists

**Highlights**
“Positive feedback from the few customers who allowed themselves to be tested.”
“Getting to know the client better, because it's kind of a personal issue, and then there was a lot of openness from the clients.”
“Client retention.”
“It was motivating that most of the clients were very open to the service.”
“I also found it great to deal with a new service that has nothing to do with Covid19.”
“The exchange and training with PCN-S was interesting.”
“Good conversations with clients! Clients reacted positively to this service.”
“One positive case.”
“Uncomplicated and easy to implement new service which was appreciated by almost 100% of the clients contacted.”
“Not much paper needed to carry out the study.”
“It is fast, easy and interesting.”
“Free screening.”
“The possibility given to the pharmacy to possibly find patients with hepatitis C.”
“Free test.”
**Weaknesses**
“Little time to spare.”
“It remains a sensitive issue for customers.”
“Rejection of substitution patients.”
“Aroused mistrust.”
“Deadlines not met on the part of clients.”
“The time of the project start coincided exactly with the start-up phase of the Covid self-test dispensing and accordingly the customer volume was enormous during this time, so that unfortunately I was not able to approach as many customers as I had hoped.”
“Sometimes difficult, who to approach?”
“Little response to invitation.”
“Additional time expenditure, although well feasible when planned for.”

## Discussion

In our assessment of the feasibility and pharmacists’ acceptance of a pilot HCV screening service in community pharmacies, 25 Swiss community pharmacies addressed 435 clients, of which 145 (33%) were interested in having a screening performed.

Having 8 of 145 screenings return positive test results, as reported back to the study group, amounts to a positivity rate, or prevalence, of 5.5%. Given an estimated HCV prevalence of 0.5% for the general Swiss population [11], addressing vulnerable persons in community pharmacies appears to have been purposeful. Our results are comparable to the dry blood spot testing service in community pharmacies on the Isle of Wight, which identified 13 new patients with HCV in 186 performed tests (7.0%) [21]. Radley and colleagues conducted a quantitative service evaluation to assess feasibility and scalability of dried blood spot testing for HCV comparable to ours. Of 143 opioid substitution users that were addressed for testing in pharmacies, 43 (30%) agreed to participate. This is comparable to our questionnaire results, where 30.7% agreed in the overall addressed population and 34.7% agreed in the subset of drug users. Radley and colleagues’ testing, however, returned 12 of 43 (28.0%) positive, which is a higher positivity rate than ours (5.5%) [31]. This difference may be explained by the focus on opioid substitution users, with previous or active drug users being considered at the highest risk for HCV infection [32]. Current models for Switzerland estimate HCV prevalence in people who use drugs between 25 and 40% [18].

Addressing clients in Switzerland due to their vulnerability for HCV including certain behaviours and sexual orientations [13, 17], has the potential to foster preconceptions and dismissive reactions. Three of the five reported barriers for pharmacists likewise concerned HCV being a sensitive topic. Concurrently, 78.6% [11] of the responding pharmacists stated that their clients wanted to use the screening when approached (64.3% rather does apply, 14.3% does fully apply), a possible indicator that the clients, once approached, reacted positively. Addressing vulnerable populations is, finally, just a triage system based on evidence on local infection sources as proposed by the Swiss Federal Office of Public Health. However, addressing these personal vulnerabilities will require strong communication skills from community pharmacists. This is required from a profession, which is described by some to “use technical jargon, display feeble responses to emotional prompts, and control the interaction and content by using close-ended questions” [33].

Two of the main selected facilitators concerned financial items: being able to offer a rapid test free-of-charge to the client and the pharmacy was mentioned by 73.3% [11] of responding pharmacists, and having the service remunerated was stated by 40.0% [6]. This was also reflected in mentioned highlights such as “Free screening.” We argue that this is representative of two difficulties in modern pharmaceutical care: pharmacists struggling with charging clients for services instead of products and pharmacists longing for remuneration for their cognitive services. If national HCV elimination strategies were to include screening interventions by community pharmacies, clear guidelines for billing and remuneration would need to be established. We, additionally, argue that at least parts of the strategy for the SARS-CoV-2 pandemic could be adopted, where Swiss pharmacies were also involved in screening and certain populations either had to pay for the test themselves (e.g., travellers) or the costs were reimbursed by the Swiss government [34].

Our pilot may have also benefitted from community pharmacies being part of the Swiss efforts battling the SARS-CoV-2 pandemic in other ways. Setting up appointments, performing swabs of the oral mucosa, carrying out rapid tests, handling potentially infectious material, and helping clients interpret test results are all skills that were needed and extensively used for SARS-CoV-2 as well. Likewise, community pharmacies were still busy with testing and vaccinating additional clients, which may have negatively affected recruiting and response rates for this pilot.

Apparently, being able to offer yet another new service to their clients was an additional facilitator (66.7%), with stated highlights such as “The possibility given to the pharmacy to possibly find patients with hepatitis C” and “I also found it great to deal with a new service that has nothing to do with Covid19.” These statements are, again, similar to the work by Radley and colleagues, where pharmacist participating in dried blood spot testing commented “’a great opportunity’, ‘an obvious thing to do’ and ‘a no brainer’” [31]. Two additional facilitators in our evaluation concerned training and help: the training was mentioned by 66.7% pharmacists, and the offered documentation around the project by 40.0%. This once again underlines the need for comprehensive training and available documentation for future pharmaceutical HCV services.

### Impact

With this assessment of feasibility, we showed that a screening for HCV in Swiss community pharmacies could be implementable, given flexibility in provision and extensive training in communication strategies, as well as financial remuneration. A screening approach through community pharmacies is in line with the Swiss Federal Office of Public Health’s decision to target specific vulnerable populations for HCV testing, follows recent calls by Bechler & Schmassmann [35] and Bihl and colleagues [18] to increase screening for HCV in Switzerland, and is in accordance with the recommendation by the European Centre for Disease Prevention and Control guidance that pharmacies should be part of the screening process [19]. Flexibility is needed to allow for variation in when and by whom precisely the screening is offered and received, adapted to the local population. Clear and empathetic communication styles will be needed to carefully approach persons from vulnerable populations. By providing on-site training and letting the pharmacies define their own communication strategies for selected populations, our pilot allowed for this flexibility and simultaneously called for participating pharmacies to take a critical look at their individualized approach.

### Limitations

Our feasibility assessment has several limitations. Importantly, there was significant dropout in performing the service and questionnaire filling. Out of the originally involved 36 pharmacies, 25 actively participated. Of these, only 15 handed in their aggregated counts of reasons for addressing and recruiting their clients. Likewise, only 15 pharmacists answered our questionnaire on acceptability, feasibility, and appropriateness of the new service. This dropout becomes apparent when comparing the number of reported screenings: Of 145 screenings reported for pay-outs, of which eight were positive, only 79 were mentioned in the separate questionnaire. Furthermore, the number of positive rapid tests were not assessed within the questionnaires, which would have allowed for stratification by vulnerable population. As individual client data were not assessed, the proportion of clients successfully linked to additional care for diagnosis and treatment was not known. This missingness may introduce distortions in our analysis and interpretation, especially the comparisons to national HCV prevalence estimates. Pilot results consisting of only 15 responding pharmacies also have limited generalisability. It is possible that especially committed pharmacies that feel particularly comfortable with their clients have participated, which introduces a selection bias. Additionally, we queried on pre-specified barriers and facilitators to reduce questionnaire filling time during the SARS-CoV-2 pandemic. This approach may have introduced anchoring bias by influencing subsequent free text answers. The additional free text answers, however, also included independent statements, showcasing that not all respondents were influenced. Finally, being a feasibility assessment, an active comparator was not part of the investigation, which negates any comparisons to other health care service providers. Direct comparisons were, however, part of the studies in Scotland [22].

### Conclusion

This assessment showed the general feasibility of an HCV screening service with rapid saliva antibody tests in Swiss community pharmacies. Important facilitators and barriers were identified, which included the sensitivity of approaching pharmacy clients according to their vulnerability towards an infectious disease. Given appropriate communication trainings and remuneration, Swiss community pharmacies may be an important partner for achieving the HCV goals set by the WHO.

## Data Availability

The dataset generated and analysed in the current study is not publicly available due to its sensitive nature, but is available from the corresponding author upon reasonable request.

## Abbreviations

D-CH: German-speaking Switzerland
F-CH: French-speaking Switzerland
HCV: Hepatitis C virus
I-CH: Italian-speaking Switzerland
PCN-S: Pharmaceutical Care Network Switzerland
SD: Standard Deviation
WHO: World Health Organization
